# Patient and public involvement facilitators: Could they be the key to the NHS quality improvement agenda?

**DOI:** 10.1111/hex.13023

**Published:** 2020-02-05

**Authors:** Sarah Todd, Christine Coupland, Raymond Randall

**Affiliations:** ^1^ Centre for Professional Work & Society School of Business & Economics Loughborough University Loughborough UK

**Keywords:** patient and public involvement, patient and public involvement facilitators, quality improvement

## Abstract

**Objective:**

Research into patient and public involvement (PPI) has not examined in detail patient and public involvement facilitators’ (PPIFs) roles and activities. This study analysed PPIFs’ roles using qualitative data gathered from three different UK health‐care organizations.

**Design:**

Thematic analysis was used to examine cross‐sectional data collected using a mixed‐methods approach from three organizations: a mental health trust, a community health social enterprise and an acute hospital trust. The data set comprised of 27 interviews and 48 observations.

**Findings:**

Patient and public involvement facilitators roles included the leadership and management of PPI interventions, developing health‐care practices and influencing quality improvements (QI). They usually occupied middle‐management grades but their PPIF role involved working in isolation or in small teams. They reported facilitating the development and maintenance of relationships between patients and the public, and health‐care professionals and service managers. These roles sometimes required them to use conflict resolution skills and involved considerable emotional labour. Integrating information from PPI into service improvement processes was reported to be a challenge for these individuals.

**Conclusions:**

Patient and public involvement facilitators capture and hold information that can be used in service improvement. However, they work with limited resources and support. Health‐care organizations need to offer more practical support to PPIFs in their efforts to improve care quality, particularly by making their role integral to developing QI strategies.

## 1 INTRODUCTION

The meanings of patient and public involvement (PPI), and the various terms associated with it, have long been debated. There is a considerable lack of consensus in the academic community about the meaning of PPI and its purpose,^1^, ^2^, ^3^, ^4^, ^5^, ^6^, ^7^, ^8^, ^9^, ^10^, ^11^, ^12^, ^13^ with words such as ‘involvement’, ‘participation’, ‘engagement’ and ‘empowerment’ used interchangeably.^14^

Early forms of PPI were triggered by activists and lobbyists wanting more public accountability in health services, for example Community Health Councils in the 1970s.^15^, ^16^ The nature of PPI has evolved with the notion of the health‐care ‘consumer’ and quasi‐marketization during the 1980s and 1990s.^17^, ^18^, ^19^, ^20^, ^21^, ^22^, ^23^, ^24^ It has been argued that PPI encompasses a wide range of activities that can be summarized as the exercise of ‘choice’ (consumers choose which service to access), ‘voice’ (consumers say what they want from their own care and wider services) and ‘exit’ (consumers can leave if they are unhappy).^9^, ^18^, ^19^

Furthermore, following numerous NHS scandals in the 2000s and 2010s, there has been pressure to increase the patient and public voice in NHS services. For example, Lord Darzi's^25^ review cited the need to measure patient experience in equal measure to patient safety and clinical effectiveness. The Francis^26^ report into the Mid‐Staffordshire NHS Foundation Trust failings highlighted the need for patient views to be integrated more effectively into quality governance structures.

Research into PPI has mainly focused on the mechanisms of PPI, the varying perspectives on PPI and the motivations of the patients and public involved.^4^, ^5^, ^11^, ^27^, ^28^, ^29^ A large body of research focuses on PPI in health research,^6^, ^10^, ^30^, ^31^, ^32^, ^33^, ^34^, ^35^, ^36^, ^37^, ^38^, ^39^, ^40^, ^41^, ^42^, ^43^, ^44^, ^45^, ^46^, ^47^, ^48^ particularly in more recent years. However, the specific role of patient and public involvement facilitators (PPIFs) remains under‐researched. Occasionally, there are brief references to their roles in studies that focus on lay/patient perspectives.^12^, ^49^, ^50^, ^51^, ^52^ In this article, we present a qualitative study of the experiences, motivations and perspectives of PPIFs in three health‐care organizations. In doing so, we offer insights into how their role can be linked to quality improvement (QI).

## 2 METHODS

### 2.1 Setting

Three separate organizations were studied in this cross‐sectional thematic analysis research: a mental health trust (MHT), a social enterprise (SE) providing community care and an acute hospital foundation trust (AHFT). This approach was used to capture both the common and context‐specific elements of PPIFs’ roles. Each organization differed in terms of size, budget and remit. MHT employed around 6000 staff and provided a range of inpatient and community mental health services; SE employed around 2000 staff and provided community health services; and AHFT employed around 8000 staff and provided acute hospital services across two sites.

### 2.2 Design and data collection

A mixed‐method approach was used. Some of the PPI activities used were identified through conversations between the lead author and the organization's named PPI lead (identified from a Strategic Health Authority's website). PPI activities were then observed by the lead author during visits over a period of 1 year between June 2013 and July 2014, with detailed observation notes collated.

Patient and public involvement activities observed included focus groups, project groups and governance meetings (see Table 1). These activities were classified as ‘PPI activities’ because at least one layperson or patient representative was involved. Twenty‐seven interviews were conducted with health‐care managers, PPIFs, laypeople/patient representatives and health professionals (see Table 2).

**Table 1:** Observation summary

Observation type	Mental health trust	Social enterprise	Acute hospital foundation trust
Focus group		2	2
Public engagement event			2
Committee/Panel		7	3
Project group	5		3
Governance meeting^a^	11		11
Board meeting	1		1
Total	17	9	22

a NB: Social enterprise did not grant lead author access to these meetings.

**Table 2:** Interview summary

Case site	Senior managers	Senior clinicians	Managers	Support staff	Service users	Carers	Professional laypeople^a^	Total
MHT	1 (PPIF)	2	3 (2 PPIFs)	1 (PPIF)		2		9
SE	3 (1 PPIF)			2 (both PPIFs)	1	1	1	8
AHFT	2 (all PPIFs)	1	2 (both PPIFs)	1 (PPIF)	1	1	2	10

Abbreviations: AHFT, acute hospital foundation trust; MHT, mental health trust; PPIF, patient and public involvement facilitators; SE, social enterprise.

a Refers to laypeople who have more formal roles, such as Public Governors.

### 2.3 Data analysis

**Figure 1:**
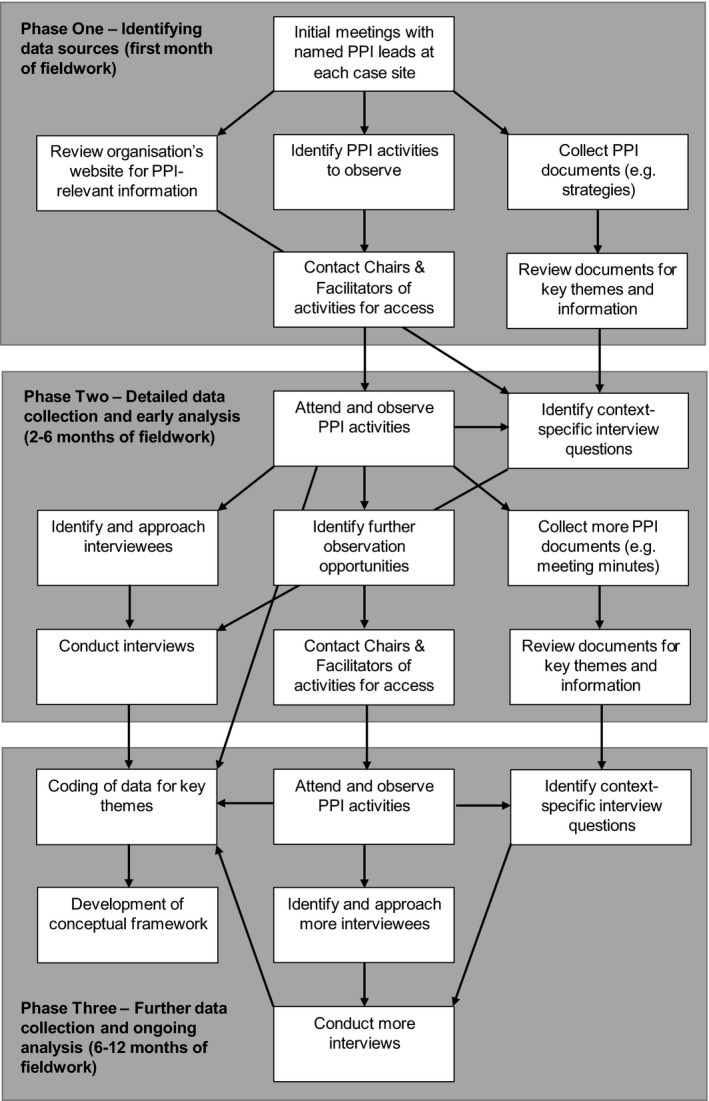
Data collection and analysis process

Thematic analysis was conducted using an iterative process of coding to identify key themes.^53^ This technique allowed for the identification of patterns both within and across sources of data. No set of pre‐defined themes was applied to the data. A variety of factors were identified during a literature review, but the analysis was inductive (see Figure 1). This allowed for more freedom to identify novel and nuanced findings.^54^

## 3 FINDINGS

Some themes which were identified resonated with established findings, such as the issue of ‘unrepresentative’ voices,^49^, ^55^, ^56^, ^57^, ^58^, ^59^, ^60^, ^61^, ^62^, ^63^ as well as numerous other factors more specific to contexts, such as practical barriers to PPI.^15^, ^27^, ^31^, ^55^, ^64^, ^65^, ^66^, ^67^, ^68^, ^69^, ^70^, ^71^, ^72^, ^73^, ^74^, ^75^ The novel findings were related specifically to the role of PPIFs and are the focus of this paper.

### 3.1 What is a PPIF?

Our study showed that a PPIF is principally responsible for acting as a link between health‐care organizations and patients and the public.^76^ They facilitate and support patient and public access to PPI activities. This ranges from providing them opportunities for giving their views through surveys and formal complaints, to securing PPI in service improvement projects, and facilitating attendance at governance meetings. These activities are widely referenced in the literature,^5^, ^24^, ^47^, ^51^, ^77^, ^78^, ^79^, ^80^, ^81^, ^82^ but with little reference to the role that PPIFs played in supporting these activities.

In interviews, many PPIFs reported that they were responsible for providing reports to senior management through governance meetings and that they supported service managers and clinicians in involving patients in service improvement projects. They interacted with a diverse range of stakeholders: current and former patients, the general public, charities, interest groups, health professionals (nurses, doctors, therapists, etc), managers and Board members.

Patient and public involvement facilitators and their colleagues referenced three main drivers for PPI work: QI (the need to continuously review and improve services); national policies or initiatives (some PPI activities such as recruiting public members were specifically required in government policy); and reputation management and transparency (the need to appear to be transparent and open to patient/public views). These drivers for PPI reportedly influenced the work PPIFs conducted, and their role within their organization. Quotes have been provided below to illustrate these rationales and the varied work undertaken by PPIFs.

Quality improvement:

I kind of see [PPI] as a tool for helping us with our patient experience agenda. So ultimately, my role is to make services better and fit for purpose, and my belief is that you can only do that by involving and listening to patients about their experience. MHT: PPIF (ID IA002)

National policies and initiatives:

When you’re a Foundation Trust…You have membership, which is drawn from members of the public…And the idea is that those members have a bit of a vested interest in those services that we provide and are able to…[give] their views on what we provide. MHT: PPIF (ID IA008)

Reputation management and transparency:

[Governors] are more like ambassadors as well because I think these times where the press is very quick to criticise the NHS, we need people out there to say…”Well actually, I’m a governor at the hospital and, in my experience, this is what I’ve been told”. AHFT: PPIF (ID IC009)

### 3.2 Formal position in the organization

According to interview data and initial conversations with PPIFs, they were usually based within central corporate functions, reporting to senior managers and the Board (directly or via their line manager). Many were members of teams of up to six people, and in AHFT, there was no specific team at all, with PPI work spread across multiple corporate functions (such as public membership office and formal complaints). As identified through interviews, these roles did not appear to confer any formal decision‐making powers. However, PPIFs’ position in the hierarchy (formal power^83^) indicated that they could advise service managers and clinicians (expert power^83^) about necessary service improvements following feedback from patients. Sometimes, this advice was not acknowledged or utilized, rendering the PPI activity an ineffective use of time and resources, as illustrated in the quote below.

‘I’d hear great ideas…but my ability to actually deliver those, because I was an engagement officer, you’re the middle man…you don’t have the power to make the change’. AHFT: PPIF (ID IC003)

Patient and public involvement facilitators’ formal positions indicated the potential for them to be involved in the management of disagreement between stakeholders. PPIFs reported that they were developing and maintaining relationships between different professional groups, who may have different perspectives. Observations of various PPI activities showed that their facilitation role brought them into contact with a wide range of stakeholders: managers (both corporate and service‐level), nursing and medical staff, professionals with specific expertise (eg chaplaincy), charitable organization representatives and laypeople. As such, a key challenge of their role was in facilitating conversations between these diverse groups.

### 3.3 Context‐specific differences

A range of context‐specific differences appeared to have an influence over PPIFs’ roles and responsibilities, and the way in which they perceived support for PPI in their organization.

#### 3.3.1 Practical delivery of services

Both MHT and SE had services spread across multiple sites including in health centres and small community hospitals, meaning they had a physical presence in the community. In contrast, AHFT was primarily based on one hospital site. PPI activities often involved laypeople coming to the hospital, rather than PPIFs going out to the community. The physical space within which these activities took place was, therefore, very different. PPIFs in MHT and SE went out to communities rather than requiring the public to come to them. This difference is highlighted in the quote below:

I always remember people, when they [patients/public] talk about the hospital, although it’s very highly regarded as a good hospital, it’s a bit of an ivory tower. Even its geographical sense…gave it the whole “a hospital is something you go to.” Which is, I think, something for most acute hospitals…So there’s an expectation that maybe you wouldn’t connect with your hospital. AHFT: PPIF (ID IC003)

#### 3.3.2 Cultural differences

Staff in both MHT and AHFT expressed a view that mental health providers are better at PPI and cited this as being as a result of an organizational culture that sits more comfortably with the principles of PPI. In acute hospital care, the patient is often seen quickly to treat a physical problem and then sent home as soon as possible, limiting the time the patient spends with staff. In a mental health setting, the relationship between health professionals and their patients is generally much longer. Many mental health patients will be accessing those services for the rest of their lives. As such, greater partnership working occurs at the clinician‐patient level, arguably making PPI a more natural approach for mental health staff than acute hospital staff. This is demonstrated in the two quotes below.

But you would expect a mental health clinician to be very good at listening to service users and carers by the very nature of their work. MHT: Manager (ID IA003)

I was talking to people from the mental health trust. And, oh my god, I was just so jealous that they get it…They realise that the only way to change behaviour and improve health is through an engagement model, investing in it. AHFT: PPIF (ID IC003)

#### 3.3.3 Organizational strategy

All three organizations had small teams responsible for PPI, but a key difference was noted in AHFT which had no specific team or budget allocated to PPI. Both MHT and SE had a designated PPI lead, whereas there was no overall lead at AHFT. Both MHT and SE also had a PPI strategy document. As such, there seemed to be less coordination of PPI activities in AHFT. The primary focus for AHFT appeared to be managing formal complaints and meeting targets (ie ‘transactional’ functions). A lack of strategic direction was cited as problematic by some staff interviewed at AHFT (example below).

Oh, and the other thing was we never had a strategy [rolling eyes]. I don’t think I’ve ever seen a strategy…[senior staff] have very different opinions about what its purpose was so it never got signed off…So there was no direction on what’s our purpose…what do we really want to get out of engagement? AHFT: PPIF (ID IC003)

### 3.4 Multiple identities within the PPIF’s role

Patient and public involvement facilitators appeared to operate in three distinct but overlapping roles: mediator, negotiator and gatekeeper. PPIFs described themselves, and were also observed to operate, in dual positions of employee/professional and patient/public representative. This resonates with a finding by Li *et al* 84 whom reported that public involvement practitioners identified themselves as ‘trusted advisor to the organizational leadership [as well as] champion for community residents’ (p. 17).

#### 3.4.1 Gatekeeper

In this role, they were determining laypersons’ access to PPI activities. They appeared to hold informal power pertaining to the nature of the access laypeople were granted. A large part of this role was in building and maintaining relationships between stakeholder groups. PPIFs were usually the primary contact for patient and public representatives and groups. As such, the access they have is at least initially determined by the PPIFs. Tenbensel^52^ noted that there are individuals providing access to decision‐makers in health policy. These individuals are integral to aiding decision‐makers’ interpretations of the public input. In this study, PPIFs appeared to play the same role in their organizations. Gibson *et al* 20 refer to these individuals as ‘salaried involvement professionals’ (p. 534) whose role is to facilitate access to PPI activities.

What I’ve done is make sure that I’ve kept in contact [with community leaders], that I’ve shared information that I feel they need to know. If ever they’ve had a question, even if it’s something I can’t deal with, I’ve encouraged them to come to me and I’ve made sure I’ve got the answers. So, it’s been about building trust. MHT: PPIF (ID A002)

#### 3.4.2 Mediator

Patient and public involvement facilitators frequently facilitated conversations between staff and patients/the public in arenas such as governance meetings or committees. These interactions often led to heated debate, as observed by the lead author and reported by PPIFs in interviews. As such, PPIFs were central in mediating that conflict and ensuring the various stakeholders were able to make a fair contribution to conversations, as illustrated in the quote below.

Well, it’s a challenge [to chair a meeting]! [laughing] I think, one, because of the size of the group. …But then I think, two, because it’s members of the public, so to speak, they kind of don’t feel so restrained as they would be if they were sort of in a professional capacity. And sometimes they’re not always used to the kind of protocols of meetings…So yeah, it can be challenging SE: PPIF (ID B008)

#### 3.4.3 Negotiator

Patient and public involvement facilitators reported having to use negotiation skills to encourage managers and health‐care professionals to 1) consider involving patients and the public in their service improvement projects and 2) implement changes based on that PPI work or other patient feedback. For example, a PPIF at MHT reported managing to do some PPI work just in time to influence a major service change that would have considerably impacted on those patients. The managers had, according to the PPIF, changed their original course of action as illustrated in the quote below.

I think the best one we’ve done so far was on a really emotive subject and that was the psycho‐oncology service, which is where we were proposing some changes [including the removal of a senior specialist]. It was quite emotive and lots of angry patients…Actually, the views of the patients completely changed what they were planning to do. They still got the same outcome that they were looking for [saving money] but they did it in a way that still met the patients’ needs. MHT: PPIF (ID A002)

### 3.5 Tensions that limited PPIFs’ roles

Data analysis identified three tensions influencing PPI processes and the relationships between major stakeholders: top‐down vs bottom‐up management; individual vs collective needs; and patient experience vs patient involvement.

#### 3.5.1 Top‐down vs bottom‐up management

Many of those interviewed indicated that they believe PPI is most effective when it involves front‐line staff and/or service managers (ie those in organizations who are responsible for delivering services). This is similar to findings that PPI works best at a ‘grass roots’ level.^27^, ^64^, ^85^, ^86^ All PPIFs in our study suggested that greater front‐line staff engagement was needed in PPI processes. PPIFs felt they had to lead on all PPI work because they believed the workload and its complexity placed too many burdens on front‐line staff.

And you know, we feel…we own it more. If there was any way of getting them and the divisions, clinical staff, to own it just that little bit more, that might help [its success]. MHT: PPIF (ID A009)

Some PPIFs reported that the lack of engagement of front‐line staff may partly be because PPI had been labelled within corporate roles (see quote below). Some felt that front‐line staff disengaged because they believed PPI was the responsibility of someone else. This is in keeping with Fudge *et al* 87 who found PPI was led by a small number of individuals in corporate roles.

I could see there could come a time…where my role doesn’t necessarily need to exist. Because…to name a lead on something, often, therefore, people don’t feel it’s their responsibility. AHFT: PPIF (ID C002)

Patient and public involvement facilitators were involved in the management of on‐going tensions between the perceived advantages and disadvantages of conducting PPI centrally (top‐down or ‘centralization’)^88^ and locally (bottom‐up or ‘decentralization’).^88^ PPIFs reported being frustrated by the difficulties they encountered in their efforts to engage front‐line staff. Their view was that PPI would work best through a participatory bottom‐up approach but that this was difficult to initiate and maintain. Decentralization can, in theory, motivate front‐line staff to take on these responsibilities by giving them greater autonomy,^88^ and this appeared to be the argument presented by the PPIFs in this study.

#### 3.5.2 Individual vs collective needs

There was also evidence of tensions between individual stakeholders’ needs and the perceived collective needs of the organization. The issue of ensuring representativeness in PPI was highlighted as problematic by many professionals, including the PPIFs themselves, and is supported by wider literature.^49^, ^51^, ^55^, ^56^, ^57^, ^58^, ^59^, ^60^, ^61^, ^62^, ^63^ Interviewees were aware of the potential for laypeople to have their own agendas. There were some occasions where PPIFs had to curtail discussions that they identified as being too personal. Data analysis suggested that personal stories were valued but had the potential to detract from the core objectives of PPI, as indicated in the two quotes below.

there’s a fear sometimes…that they’re going to bring personal experiences to the table. And again, it’s a balance, because you do want people to bring personal experiences; [but] not going into the detail. SE: PPIF (ID B002)

We’ve got to set out the rules of engagement so it doesn’t become personal. So that it actually is thinking in the wider look at the service provision rather than the individual service provision. AHFT: PPIF (ID C002)

Patient and public involvement facilitators indicated that giving individuals a voice for their views, beliefs and experiences (ie giving them a form of referent power^83^) was a core task within their role. They described how they were often managing conflicts between laypersons’ priorities and resources of the stakeholders in the organization. PPIFs reported that laypeople expected their individual viewpoints to be important enough to influence service improvement. However, PPIFs were sometimes working with the knowledge that individuals’ views may be of little or no value to service managers and health professionals. This is consistent with Croft *et al* 89 who concluded that there was a risk of managers having full control over decision making by marginalizing patients’ individual perspectives because of conflict with organizational priorities. Gibson *et al* 50 go further in suggesting that PPIFs will not push patients’ views onto other staff as they would not want to ‘over antagonize’ NHS managers (p. 534), as they rely on the organization for their livelihood.

#### 3.5.3 Patient experience vs patient involvement

The interchangeable use of the terms ‘patient experience’ and ‘patient involvement’ created confusion for stakeholders about the purpose of PPI. In Arnstein's ladder of participation,^90^ PPI methods are defined according to levels of involvement, with co‐design and co‐delivery of services at the top of the ladder and providing information and tokenistic involvement at the bottom. PPIFs expressed a desire to use a variety of methods but with a preference for those nearer the top of the ladder. They felt that surveys could not accurately be labelled as ‘involvement’ but rather a measure of patient experience.

In AHFT, some PPIFs suggested that there was pressure from senior management to report against KPIs and statistics by conducting surveys and managing complaints, rather than what they deemed to be ‘proper’ involvement (see quotes below). Some PPIFs reported that front‐line staff would sometimes conduct surveys and describe this as ‘patient involvement’, therefore ‘ticking the box’ on their obligation to involve patients in service changes. PPIFs had a more nuanced interpretation of PPI and appeared to value different PPI methods in hierarchical terms, much like Arnstein's ladder of participation.^90^ They suggested surveys would be at the bottom of the ladder and would prefer to aim for more involvement of patients and the public in decision‐making processes.

All we do is a patient satisfaction, and in fact, we’re going more that way than proper engagement because I’ve seen it move towards numbers…so we’re doing the low‐level engagement on “are we doing a good service?” We’re not even doing the level above that – the “what would make a better service?”…not anything more engaging. AHFT: PPIF (ID C003)

Patient and public involvement facilitators also commented that senior management and front‐line staff did not always fully comprehend the breadth and depth of PPI activities and the potential benefits (see quote below). Some indicated that this was because of a lack of deep understanding of PPI theory and practice. Moreover, some PPIFs felt that PPI knowledge was tacit and thus difficult to transfer.

Sometimes I feel that they [the Board] don’t always quite understand what’s actually involved in it…I hear them talk about the patient experience programme, they’ll get the name wrong and you’ll think “Ah, you don’t really understand”. MHT: PPIF (ID A009)

#### 3.5.4 How these tensions impact on PPIFs

There was evidence that the tensions between top‐down and bottom‐up management were influencing decision‐making processes and power relationships. PPIFs regularly needed to simultaneously obtain the support of senior management for strategic organizational change, as well as the support of service managers and front‐line staff at the local level. The frequently reported and observed tension between individual and collective needs was also a considerable challenge for PPIFs to balance when it came to recommending service improvements. Senior managers needed to manage both individual and community‐wide needs simultaneously. Whenever an individual's needs conflicted with wider organizational priorities, the latter prevailed.

**Figure 2:**
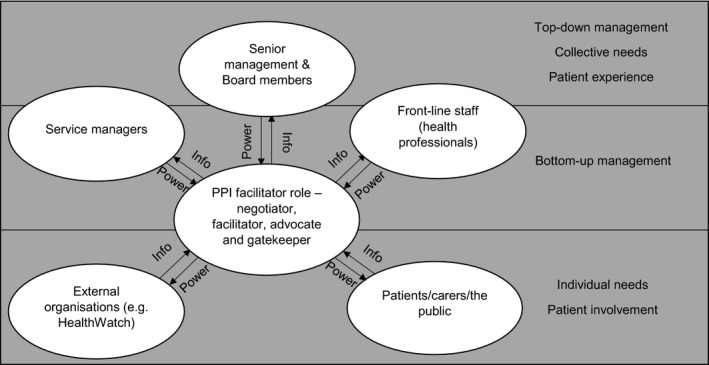
Patient and public involvement (PPI) Facilitator role conflicts and tensions

The influence of these tensions is illustrated in Figure 2. PPIFs were often reduced to simply sharing information with senior managers and service managers in the hope that it would lead to service improvements, but not having the power themselves to enact changes.

### 3.6 The mirrored characteristics of laypeople and PPIFs

There were similarities between the reported motivations, beliefs and characteristics of PPIFs and those of the laypeople involved in PPI. For example, all laypeople had experienced or witnessed poor care, and this reportedly motivated them to take part in the PPI process. During interviews, they often claimed to be motivated by wanting to give other people a voice and improve services as a result. These experiences and motivations seemed to be shared by the PPIFs.

**Figure 3:**
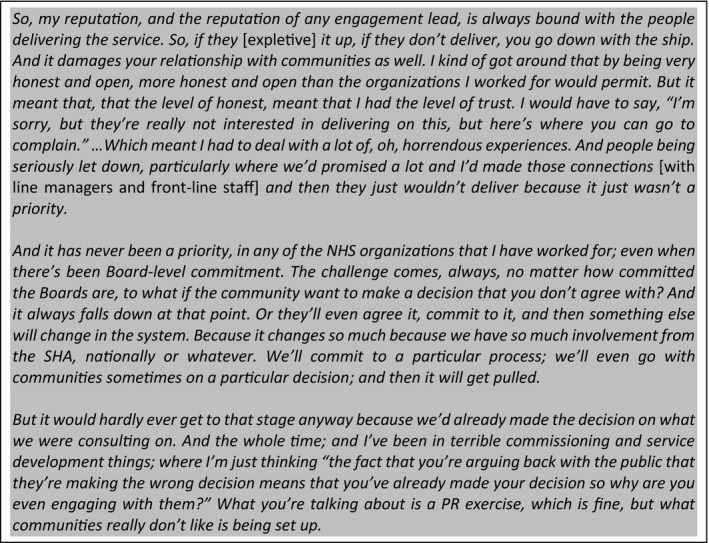
Vignette from a patient and public involvement facilitators (PPIF)AUTHOR: Figure 3 has been saved at a low resolution of 116 dpi. Please resupply at 600 dpi. Check required artwork specifications at https://authorservices.wiley.com/asset/photos/electronic_artwork_guidelines.pdfReplacement figure supplied via email in PDF format

Barnes *et al* 85 found similar activist histories and traits in the PPIFs they interviewed, an important finding for the future understanding of PPI practices. This evidence suggests that PPIFs’ allegiance could be most strongly aligned with patients and the public, rather than their employer. To illustrate the conflicts and challenges a PPIF experiences in their role, Figure 3 shows a vignette from a PPIF working in AHFT.AUTHOR: Barnes et al. found to be mismatch with this reference citation [81], please check.Reference should be number 85

## 4 DISCUSSION

This research adds to the literature by examining the unique relationship between patients and the public and PPIFs, as well as between PPIFs and their colleagues. Many of the colleagues PPIFs engage with are from established professions. As a result, it may be challenging for them to identify with these individuals, which may lead to an internal conflict concerning whom they identify most closely with.

The findings also indicate the potential for power conflicts to develop. Health professionals and managers may view PPIFs’ motivations as most closely aligned with patients and the public, leading to a distrust of PPIFs. Furthermore, Bolton^17^ made a suggestion that using feedback from complaints is merely a tool for monitoring and performance managing staff, something also suggested about wider PPI by Milewa *et al* 80 If health professionals view PPIFs as using PPI to performance manage them, this may create further conflict. In this study, PPIFs reported that some front‐line staff and service managers resisted being involved in PPI initiatives. One of the reasons for this resistance could be their perceptions of PPIFs’ motivations and allegiances with patients and the public.

Furthermore, adding to these conflicts of legitimate, expert and referent power^83^ was the prevalence of an ‘in‐group’. There was a limited pool of people who PPIFs invited to join projects, groups and committees. This resulted in the same group of people being included in multiple activities. The finding supports research that has noted this ‘in‐group’ phenomenon in PPI.^55^, ^59^, ^86^, ^91^ This potentially could exacerbate power conflicts with health professionals and managers who may argue that this type of group is unrepresentative, therefore justifying questioning PPIFs’ recommendations.

Laypeople defined their own role as a result of being motivated by seeing or experiencing poor care. This confirms findings from existing literature on layperson motivations and identities.^32^, ^49^, ^63^, ^68^, ^79^, ^92^, ^93^, ^94^ In addition, the PPIFs identified themselves as advocates more than employees. This study indicates that often the motivations and identity perspectives of laypeople were mirrored by PPIFs.

These conflicts and the complexity of the PPIFs’ roles appeared to be very challenging and involved considerable emotional labour and cognitive workload, as they often worked in isolation, as reported by Staniszewska.^12^ One of the major recommendations for future practice is to improve support for PPIFs in their daily work.

Patient and public involvement facilitators indicated that their role was constrained by its level within the organization, in that they neither had the power to make decisions nor did they have the power to enact service changes. It was only within their power to try to influence service changes through sharing intelligence. Figure 2 shows the groups of stakeholders PPIFs are required to work with and how information is exchanged or brokered by PPIFs. It highlights that actual power for decision making (and therefore service changes) rests with service managers and senior clinical staff, and/or senior management.

### 4.1 Implications for practice

The main recommendations from this study focus on supporting PPIFs to balance the highlighted tensions. As PPIFs are key in all PPI processes, from being a gatekeeper for patients and the public, to facilitating stakeholders’ conversations, to making recommendations for service improvements, it appears important to provide them with adequate instrumental and emotional support. These individuals may begin to feel ineffective and under‐valued if these are not provided.

As there was evidence that PPI was not embedded in decision‐making processes, senior managers may need to be clearer about the value of PPI in their organization. If the purpose is not clear, or PPI conflicts with wider organizational priorities, it is unlikely to improve services. In order to prevent PPI conflicting with organizational strategy, we suggest PPIFs should be involved in strategic decision making. Furthermore, PPI activities should be aligned with said priorities so that it directly feeds into organizational decision making, for example by involving patients in developing cost‐saving initiatives when under financial pressure.

Patient and public involvement facilitators should also be supported in more practical and tangible ways, such as through training and mentoring, as well as through ensuring access to adequate financial and physical resources. This has been suggested elsewhere in the context of PPI in research.^95^ This could facilitate greater variance of PPI activities, including more targeted work with hard‐to‐reach groups, and the ability to hold activities in multiple venues.

### 4.2 Study limitations and future research

A limited number of PPIFs were interviewed from three health‐care organizations. As such, we cannot be confident that the perspectives of the PPIFs presented here would be found in other organizations. As Barnes *et al* 49 suggested, PPIFs’ motivations, beliefs and perspectives should be given equal consideration with those of the patients and public. We propose that further research is carried out which focuses on PPIFs’ perspectives. This could be done through surveys, in‐depth interviews and/or focus groups, in order to gain both rich insight and patterns of personal engagement.

As there were conflicts between PPIFs, front‐line clinicians and service managers, it may also be pertinent to assess the perspectives of staff not closely associated with PPI work. This would give greater insight into why PPIFs encounter barriers with these stakeholders and provide further recommendations for practice in the future.

Another potential limitation of the study may have been that, due to a lack of clear definitions within the literature regarding the specific role of PPIFs, and indeed the nature of PPI itself, this may have inadvertently biased the conclusions made. Further research may indeed lead to different conclusions about the role of PPIFs or at least add nuanced knowledge.

## 5 CONCLUSION: WHAT CAN PPIFS CONTRIBUTE TO QI?

This study has demonstrated how important PPIFs are in PPI processes. With some exceptions,^12^, ^49^, ^50^, ^52^ there is a considerable lack of acknowledgement and appreciation of the role of PPIFs in the literature. Our results indicate that PPIFs can feel isolated in their roles when attempting to influence organizational cultures and conduct QI. There was evidence that they felt that senior managers did not appreciate the benefits of PPI in general but also their specific role in PPI processes. PPIFs’ sense of isolation was further exacerbated by this lack of shared understanding and appreciation of PPI with others.

Our study also identified the difficulties that PPIFs face in balancing tensions that impact on their work. This balancing role further demonstrates how PPIFs are potentially an underutilized resource in health‐care organizations. These three tensions (top‐down vs bottom‐up management, individual vs collective needs and patient experience vs patient involvement) have implications for the QI agenda. Therefore, it is argued that health‐care organizations need to offer more support to PPIFs to ensure that their valuable contribution can be realized.

Finally, as QI was an explicitly cited driver for PPI, one would expect PPI interventions to influence service developments. The tensions underlying PPI work can inhibit PPI directly influencing QI. In addition, the power conflicts between stakeholders and PPIFs limit the ability of PPIFs to influence QI, as decision making primarily lies within the remit of managers and clinical teams. Incorporating the role of PPIFs into decision‐making processes may help them to better influence QI.

## CONFLICT OF INTEREST

The authors declare no conflicts of interest.

## Data Availability

The data that support the findings of this study are available from the corresponding author upon reasonable request.
